# Selenomethionine Ameliorates Cognitive Impairment, Decreases Hippocampal Oxidative Stress and Attenuates Dysbiosis in D-Galactose-Treated Mice

**DOI:** 10.3390/antiox11010111

**Published:** 2022-01-04

**Authors:** Ying Gao, Yongquan Xu, Junfeng Yin

**Affiliations:** Tea Research Institute Chinese Academy of Agricultural Sciences, Ministry of Agriculture, Hangzhou 310008, China; yinggao@tricaas.com

**Keywords:** selenomethionine, neurotransmitter, D-galactose, oxidative stress, gut microbiota

## Abstract

The prevalence of age-related cognitive impairment is increasing as the proportion of older individuals in the population grows. It is therefore necessary and urgent to find agents to prevent or ameliorate age-related cognitive impairment. Selenomethionine (SeMet) is a natural amino acid occurring in yeast and Brazil nuts. It mitigates cognitive impairment in an Alzheimer’s disease mouse model, however, whether it works on age-related cognitive impairment remains unknown. In this study, SeMet significantly improved the performance of D-galactose-treated mice in the novel object recognition test, passive avoidance task and Morris water maze test. SeMet reversed D-galactose-induced reduction of hippocampal acetylcholine levels, suppression of choline acetyltransferase activity and activation of acetyl cholinesterase. It decreased D-galactose-induced oxidative stress and increased the selenoprotein P levels in the hippocampus. Besides, it attenuated D-galactose-induced dysbiosis by increasing the α-diversity and modulating the taxonomic structure. Correlations between certain taxa and physiological parameters were observed. Our results provide evidence of the effectiveness of SeMet on ameliorating D-galactose-induced cognitive impairment and suggest SeMet has potential to be used in the prevention or adjuvant treatment of age-related cognitive impairment.

## 1. Introduction

Selenium is an essential micronutrient for animals and humans. Selenomethionine (SeMet) is the predominant active component in selenium supplements. It is a natural amino acid occurring in yeast and certain plants (e.g., Brazil nuts) [1]. Humans and animals are not capable of synthesizing SeMet but are capable of catabolizing it. Part of SeMet is converted into selenocystine and used for selenoprotein synthesis. Part of it is incorporated non-specifically into proteins as tRNA^Met^ cannot discriminate between methionine and SeMet [2]. It allows selenium to be stored in the body and reversibly released by normal metabolic processes. Due to this, SeMet is more efficient in increasing the selenium level in the body than selenocystine and the average whole-body half-life of SeMet in humans is much longer than that of selenite [3]. Dietary administration of SeMet effectively activates the expression of genes encoding selenoproteins, while it shows no sign of inducing DNA damage in the intestine, gastrocnemius, cerebral cortex and liver [4].

SeMet has multiple health benefits. Antioxidant activity is one of its characteristic activities. SeMet enhances the antioxidant capacity of human retinal pigment epithelial cells by increasing intracellular glutathione [5]. It protects human keratinocyte HaCaT cells from UVB-induced damage by decreasing oxidative stress and activating antioxidases [6]. Oral administration of SeMet potentiates free radical scavenging activity and improves glutathione and thioredoxin systems in the serum and liver [7]. More recently, studies demonstrated that SeMet reduces oxidative damage to exhibit protective effects in glutamic acid-exposed HT22 hippocampal neuronal cells [8] and attenuates cognitive impairment in lead-loaded rats [9].

Aging is a process characterized by the progressive loss of tissue and organ function [10]. Oxidative stress is associated with age-related cognitive decline [11]. However, whether SeMet attenuates cognitive impairment in aging animal models was not clear. To investigate the effect of SeMet on age-related cognitive impairment, a D-galactose-induced aging mouse model was applied. D-Galactose accelerates the aging process in various tissues in rodents and successfully mimics the natural aging process [12,13]. Therefore, the D-galactose-induced aging mouse model is a consolidated model for studying aging. The novel object recognition test, passive avoidance task, and Morris water maze were carried out to assess the cognitive performance. The content of acetylcholine (Ach), a well-known neurotransmitter for supporting cognitive function, and the activities of its key metabolic enzymes in hippocampus were measured. The levels of oxidative stress and selenoproteins were also determined. In addition, the effect of SeMet on gut microbiota, which participate in modulating the functions of the central nervous system, was also monitored. The results could help us better understand the impacts of SeMet on cognition and perhaps provide a novel prevention strategy for aging-related cognitive impairment.

## 2. Materials and Methods

### 2.1. Reagents

SeMet was purchased from Shanghai Yuanye Bio-Technology Co., Ltd. (Shanghai, China). D-Galactose was purchased from Sigma-Aldrich Chemical Reagent Co., Ltd. (St. Louis, MI, USA).

### 2.2. Animals and Experimental Design

Thirty-six two-month-old male Kuming mice were purchased from Beijing HFK Bioscience Co., Ltd. (Beijing, China) and kept in a standard environment (relative humidity of 50 ± 10%, temperature of (22 ± 2) °C, and 12-h dark/light cycles), with free access to a standard rodent diet and water. After a one-week acclimatization period, the mice were randomly and evenly divided into three groups, including a control group (CK group), a model group (M group) and a SeMet group. Mice in the M group and SeMet group were treated with daily subcutaneous injections of D-galactose at a dosage of 100 mg/kg body weight for 8 weeks, while mice in the CK group were treated with the same amount of saline. Each mouse in the SeMet group was administered 30 μg of SeMet per day via intragastric gavage for 8 weeks, while mice in the CK group and M group were administered the same amount of distilled water. The dosage of SeMet was set based on a previously published reference with some modifications [14]. In the reference, mice were treated with 6 μg/mL Se-Met in their drinking water. The average water intake of a mouse was 3–5 mL per day [15]. Therefore, the dosage of SeMet was set at 30 μg per day for each mouse in the present study. The animal experiment was approved by the Ethics Committee of Institute of Medicinal Plant Development, Chinese Academy of Medical Sciences (SLXD-2018041513). All animals received humane care according to the Guide for the Care and Use of Laboratory Animals.

### 2.3. Novel Object Recognition Test

The novel object recognition test was carried out on the 1st–4th day of the 8th week, according to Lueptow’s method with some modifications [16]. The test consisted of three sessions, i.e., habituation, training session, and testing session. For habituation (Day 1–2), each mouse was removed from its home cage, placed in the middle of an empty experimental chamber, and allowed to freely explore 5 min/day for 2 days. In the training session (Day 3), two identical objects were placed on opposite sides (left/right) of the chamber. Each mouse was removed from its home cage and placed in the middle of the experimental chamber with its back towards the objects. The distances from the mouse nose tip to the two objects were the same. The mouse was allowed to freely explore for 5 min. In the testing session (Day 4), one object was the same object used in the training session and the other object was replaced with a novel object which had the same size and color to that of the previous object but a different shape. Each mouse was removed from its home cage and placed in the middle of the experimental chamber with its back towards the objects. The distances from the mouse nose tip to the two objects were the same. The mouse was allowed to freely explore for 5 min. The exploration time for familiar object (Tf) and the exploration time for novel object (Tn) were recorded and analyzed using the SuperMaze software (Shanghai XinRuan Information Technology Co., Ltd., Shanghai, China). The discrimination ratio represented recognition memory sensitivity. It was calculated using the following formula: Discrimination ratio = (Tn)/(Tn+Tf). A discrimination ratio value closer to 1 indicated that mouse remembered the familiar object and preferred to spend more time exploring the novel object due to its nature.

### 2.4. Passive Avoidance Task

The passive avoidance task was carried out on the 5th–6th day of the 8th week based on Park’s method with some modifications [17]. The test had three sessions, including habituation, training session, and testing session. For habituation (Day 1), the mouse was placed to the lit compartment of the experimental chamber with its back towards the dark compartment of the chamber and allowed to freely explore both compartments for 3 min. In the training session (2 h after habituation), the dark compartment was electrified. The mouse was placed to the lit compartment with its back towards the dark compartment and allowed to explore for 5 min. If the mouse stepped into the dark compartment, it would receive a mild foot shock. If the mouse didn’t step to the dark compartment within 100 s, it was gently driven to the dark compartment. On testing session (Day 2), the dark compartment was electrified. The mouse was placed to the lit compartment with its back towards the dark compartment and allowed to explore for 5 min. The first time the mouse entered the dark compartment (passive avoidance latency) and the times it entered the dark compartment (error times) were recorded. If the mouse didn’t enter the dark compartment during the 5 min, the passive avoidance latency was recorded as 300 s and error times was recorded as 0.

### 2.5. Morris Water Maze

The Morris water maze was carried out on the 7th week, based on Vorhees’s method [18]. The mouse was placed in a circular pool with its head towards the wall. The pool (diameter of 120 cm) had four equidistant marks on the wall to quarter the pool and was filled with water (temperature of (23 ± 2) °C). A platform (diameter of 10 cm) was submerged below the water’s surface in the center of the first quadrant. The mouse was given the task to swim to the platform. If the mouse couldn’t find the platform within 90 s, it would be guided to the platform and stayed on the platform for 20 s. Each mouse was trained once per day for 5 days. On the 6th day, the platform was removed from the pool. The mouse was placed in the circular pool at the same site with its head towards the wall. The times of the mouse crossed the platform (crossing times), the target quadrant duration, and the first time that the mouse crossed the platform (escape latency) were recorded and analyzed using the SuperMaze software.

### 2.6. Sample Collection

Mice were sacrificed at the end of the 8th week. The hippocampus was carefully isolated and immediately stored at −80 °C. The intestinal content was collected by transversely cutting the lower tract of cecum and squeezing it out to a sterile microtube, and stored at −80 °C.

### 2.7. Measurements of Acetylcholine, Acetyl Cholinesterase, Choline Acetyltransferase, and Monoamine Oxidase

The hippocampus was homogenized with pre-cooled normal saline (*w*:*v* = 1:9) and centrifuged at 2500 g for 10 min. The supernatant was collected for the measurements. The content of Ach, and the activities of acetyl cholinesterase (AChE), choline acetyltransferase (ChAT), and monoamine oxidase (MAO) in hippocampus were determined using commercial kits (A105-2-1, A024-1-1, A079-1-1, and A034-1-1, respectively) obtained from the Nanjing Jiancheng Bioengineering Institute (Nanjing, Jiangsu, China).

### 2.8. Determination of Oxidative Stress in the Hippocampus

The hydrogen peroxide (H_2_O_2_) level was measured using a commercial kit (Product No. S0038, Beyotime Biotechnology, Shanghai, China), according to the manufacturer’s instructions.

The accumulation of advanced glycation end products (AGEs) in hippocampus was measured using a mouse AGEs ELISA kit (Product No. JL10691, Shanghai Jianglai Biotechnology Co., Ltd., Shanghai, China).

The ferric ion reducing antioxidant power (FRAP) was determined using a commercial kit (Product No. A015-1-2, Nanjing Jiancheng Bioengineering Institute).

### 2.9. Determination of Selenoproteins in the Hippocampus

The level of selenoprotein P (SelP) was measured using a mouse SelP ELISA kit (JL43599, Shanghai Jianglai Biotech).

The activity of glutathione peroxidase (GSH-Px) was measured using a GSH-Px assay kit (A005-1-1, Nanjing Jiancheng Bioengineering Institute).

### 2.10. Illumina MiSeq Sequencing of 16S rRNA Gene V3-V4 Region of Gut Microbiota

Illumina MiSeq sequencing of the 16S rRNA gene V3-V4 region of the gut microbiota was carried out exactly as formerly published [19]. To evaluate α-diversity, Shannon and Simpson indexes were calculated. To evaluate β-diversity, unweighted UniFrac distance was calculated. Differential taxa between groups were identified using the linear discriminant analysis effect size (LEfSe) analysis. Spearman’s rank correlation coefficients were used to evaluate the correlation between key genera and certain behavioral or physiological parameters. Functional profiles of gut microbiota were estimated using PICRUSt and KEGG databases. The differences in gene function between groups were compared with the Welch’s t-test using the STAMP software.

### 2.11. Statistical Analysis

Data are expressed as mean ± standard error of mean (SEM). The SPSS software (version 18.0 for Windows, IBM, Chicago, IL, USA) was used for statistical analysis. Physiological parameters were analyzed with one-way analysis of variance and a post hoc test (two-sided Dunnett’s test). Behavioral parameter, α-diversity indices, and relative abundance of certain taxa were analyzed with a non-parametric test (Kruskal−Wallis rank sum test). LEfSe analysis was performed with the nonparametric factorial Wilcoxon rank sum tests. A log LDA score higher than 3.0 was considered significant. Statistically different taxa were shown in the cladogram. *p* values < 0.05 were considered statistically significant.

## 3. Results and Discussion

### 3.1. SeMet Improves D-Galactose-Induced Cognitive Impairment

**Figure 1:**
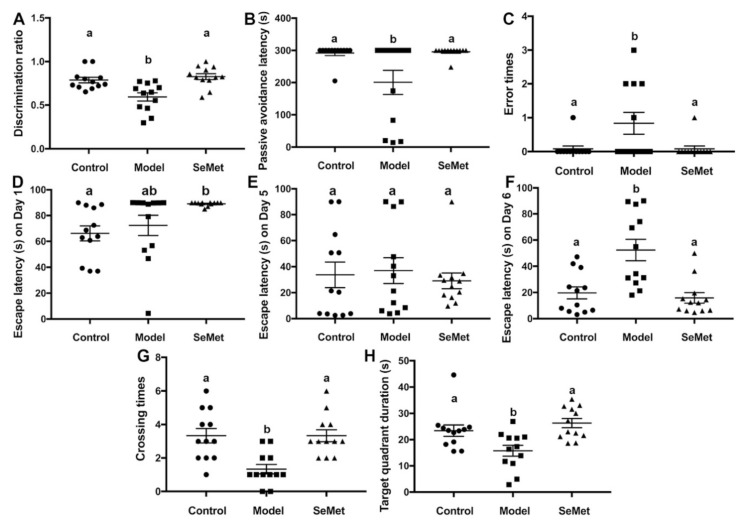
Effects of selenomethionine (SeMet) on cognitive impairment. (**A**) The discrimination ratio from novel object recognition test. (**B**) Passive avoidance latency and (**C**) error times from passive avoidance task. (**D**–**H**) Escape latency on Day 1 (**D**), Day 5 (**E**), and Day 6 (**F**), number of target crossings (**G**), and target quadrant duration (**H**) in the Morris maze test. The same letter within each column indicates no significant difference (*p* > 0.05).

The novel object recognition test is a relatively fast and efficient method to test cognition, especially recognition memory in rodents [20]. It is based on the spontaneous tendency of rodents to spend more time exploring a novel object than a familiar one. A mouse suffering from cognitive impairment is prone to get a lower discrimination ratio than a normal mouse. In this study, the discrimination ratio of mice in the model group was significantly lower compared with that in the control group, suggesting that D-galactose successfully induced cognition impairment in mice (Figure 1A). The discrimination ratio of mice in the SeMet group was significantly higher than that in the model group and showed no significant difference to that in the control group. It suggested that SeMet effectively attenuated D-galactose-induced recognition memory impairment.

The passive avoidance task is a method to measure fear-motivated memory. It involves a mouse inhibiting its behavior to avoid a shock. The mouse has to choose between one naturally aversive spot (the light chamber) and one naturally preferred (the dark chamber) but with a previously aversive experience (mild foot shock) spot. According to the results (Figure 1B,C), the passive avoidance latency of mice in the model group was significantly reduced compared to that in the control group. Correspondingly, the number of error times of mice in the model group was higher. Mice in the SeMet group were with longer passive avoidance latency and smaller error times, implying that SeMet ameliorated D-galactose-induced fear-motivated memory impairment.

The Morris water maze test is a classic behavioral test to measure spatial learning and memory. D-Galactose hampers the spatial learning and memory of mice, causing retarded escape latency, reduced crossing times of the target quadrant, and decreased target quadrant duration (Figure 1D–H). SeMet reversed the situation. The spatial learning and memory of mice in the SeMet group was not significantly different from that of mice in the control group.

Previous studies showed that the effect of SeMet on cognitive impairment was controversial. It mitigated cognitive decline in triple transgenic AD (3 × Tg-AD) mice and showed prospects in the treatment of Alzheimer’s disease [14,21]. It protected rats from lead-induced brain damage [9]. On the contrary, chronic exposure of SeMet impaired the social learning outcomes and behavior in zebrafish, and the effects were transgenerational [22,23]. Our results were supportive of the fact that SeMet ameliorated cognitive impairment in mice. A hypothesis for the inconsistent results from different studies is that the effect of SeMet on cognition might be species-specific. More studies are required to verify this.

In the study, the effect of SeMet in preventing D-galactose-induced cognitive impairment in mice was proved. Due to some limits, only one dosage of SeMet was applied. Selenium is usually known as the ‘double-edged sword element’ [24]. Adequate selenium benefits health, while excessive selenium harms health. The LD_50_ of SeMet in mice after intravenous injection was 8.8 ± 1.4 mg/kg [25]. Hence, it’s necessary to find out the safe and effective dosage range of SeMet in a future study. In addition, our results were obtained from a preventive model. The inspiring results encourage us to further investigate whether SeMet works in the treatment of age-related cognitive impairment. A former study indicated that selenium delayed the neuron injury in mice which were pretreated with D-galactose for three weeks [26]. Unfortunately, the authors didn’t mention the type of selenium they used. More detailed studies should be conducted to figure out the therapeutic effect of SeMet on age-related cognitive impairment.

### 3.2. SeMet Attenuates D-Galactose-Induced Neurotransmitter Dysregulation

Ach is an endogenous neurotransmitter used by all cholinergic neurons. It is synthesized from choline and acetylcoenzyme A by ChAT, and rapidly hydrolyzed by AChE into choline and acetate after binding to its receptors at the postsynaptic membrane. In the central nervous system, Ach is a critical modulator of cognitive functions [27]. Ach dysregulation results in cognitive impairment. Scopolamine, a competitive antagonist of Ach at muscarinic receptors, blocks the proper functions of Ach and causes amnesia [28]. The impairment of the cholinergic system is frequently observed in patients with cognitive impairment, including Alzheimer’s disease (AD) [29,30]. Many first-line medications that are currently approved for AD (e.g., galantamine and donepezil) are AChE inhibitors. They increase the Ach level in the brain by preventing the enzymatic degradation of Ach and postpones the progression of AD.

**Figure 2:**
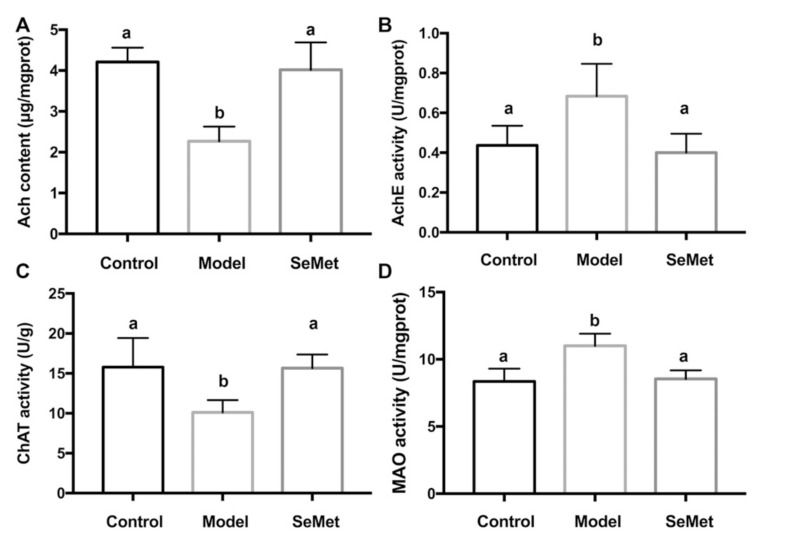
Effects of SeMet on (**A**) the acetylcholine (Ach) level, (**B**) acetyl cholinesterase (AChE) activity, (**C**) choline acetyltransferase (ChAT) activity, and (**D**) monoamine oxidase (MAO) activity in hippocampus. The same letter within each column indicates no significant difference (*p* > 0.05).

In this study, the hippocampal Ach content was significantly decreased in the model group. Along with this, the enhancement of hippocampal AChE activity and the suppression of ChAT activity were observed. This indicated that D-galactose induced Ach dysregulation in the hippocampus by interfering the metabolism of Ach. Compared with the model group, the Ach content was increased, the AchE activity was decreased, and the ChAT activity was rescued in the SeMet group (Figure 2A–C). The result implies that attenuating Ach dysregulation in hippocampus was one of the strategies of SeMet to alleviate D-galactose-induced cognitive impairment.

Besides the effect of SeMet on the modulation of Ach dysregulation, it was found that SeMet inhibited the MAO activity in the hippocampus (Figure 2D). MAO is important in the metabolism of a number of monoamine neurotransmitters [31]. The alteration of monoamine metabolism is a characteristic feature of aging [32]. Rasagiline, a MAO-B inhibitor, improves certain aspects of attention and executive functions in patients with Parkinson’s disease [33]. It also benefits clinical and neuroimaging measures in patients with mild to moderate AD [34]. Some natural products, for example, protein and anthraquinone glycosides from *Radix Polygoni Multiflori* [35], and saponins from *Liriope platyphylla* [36], decreased the MAO activity in brain and improved cognition impairment in D-galactose-induced mice. This hints that SeMet might also have an impact on regulating the metabolism of monoamine neurotransmitters.

### 3.3. SeMet Modulates Oxidative Stress and Selenoprotein Levels in the Hippocampus

Excessive oxidative stress aggravates neurodegeneration and promotes the progression of various disorders, including cognitive impairment [37]. As age increases, oxidative stress increases and oxidative stress-induced damage accumulates. An observational study revealed that increased oxidative stress was associated with age-related cognitive impairment in a healthy population [11]. Maintaining redox balance in the central nervous system forestalls cognitive impairment [38].

**Figure 3:**
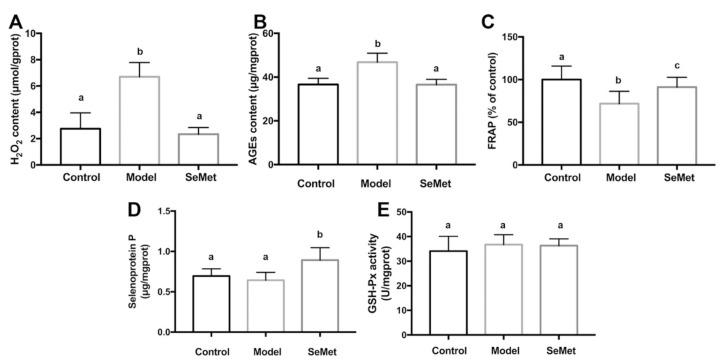
Effects of SeMet on (**A**) hydrogen peroxide (H_2_O_2_) level, (**B**) advanced glycation end products (AGEs) content, (**C**) ferric ion reducing antioxidant power (FRAP), (**D**) selenoprotein P level, and (**E**) glutathione peroxidase (GSH-Px) activity in hippocampus. The same letter within each column indicates no significant difference (*p* > 0.05).

SeMet decreased the H_2_O_2_ and AGEs levels in hippocampus of D-galactose-treated mice (Figure 3A,B). Meanwhile, it increased FRAP, a parameter which represents the antioxidant capacity (Figure 3C). H_2_O_2_ belongs to reactive oxygen species (ROS). It is generated by various cellular metabolism processes. For example, it is the by-product of MAO-mediated catecholamine metabolism [39]. H_2_O_2_ modulates synaptic transmission and contributes to neuronal damage in the central nervous system [40]. It may further react with transition metal ions to form more active ROS, such as hydroxyl radicals, and attack macromolecules [41]. AGEs are glycated proteins or lipids which are generated at an accelerated rate under hyperglycemic and/or oxidative stress conditions [42]. AGEs are important risk factors for the development of cognitive impairment in aging and neurodegenerative diseases [43]. The binding of AGEs to the receptor of AGEs (RAGE) activates the pro-inflammatory signaling pathways, increases ROS formation, and induces apoptosis in neurons [44,45]. AGEs encourage the formation and deposition of neurofibrillary tangles and amyloid plaques, which are the hallmarks of neurodegenerative diseases [46].

SeMet might lower the hippocampal oxidative stress via direct and indirect ways. Former studies proved that SeMet was capable of scavenging multiple free radicals, including hydroxyl radical, superoxide anion radical, and peroxyl radical [47,48,49]. Therefore, it was deduced that SeMet might reduce the oxidative stress in hippocampus by directly reacting and scavenging ROS. Another speculation was that SeMet decreased the formation of oxidative stress. It was reported that the inhibition of MAO led to a decreased H_2_O_2_ production in the brain [50]. In our study, it was found that SeMet suppressed the MAO activity in hippocampus. It was assumed that SeMet might decrease the hippocampal H_2_O_2_ level by inhibiting the MAO activity. Additionally, SeMet might decrease the oxidative stress by enhancing the antioxidase system. SelP is abundant in neurons and ependymal cells in the brain [51]. It plays key roles in maintaining the synaptic function in hippocampus and ameliorates oxidative stress in the aging brain [52]. Besides acting as an antioxidase itself, SelP also works as a Se transport protein, which promotes the expression of other antioxidant selenoproteins [53]. In this study, an elevated SelP level was observed in hippocampus of mice in the SeMet group (Figure 3D). Unexpectedly, the activity of GSH-Px, whose active center contains the selenium element and catalyzes the degradation of organic hydroperoxides, was not affected (Figure 3E). The results implied that SeMet might enhance the antioxidant activity of hippocampus by increasing the SelP level. Whether selenoproteins other than GSH-Px were involved in this needs further exploration.

### 3.4. SeMet Alleviates D-Galactose-Induced Dysbiosis

The gut-brain axis is the bidirectional communication between the central nervous system and the gastrointestinal tract. The gut microbiota is a component of the gut-brain axis and plays roles in cognitive development and health [54]. Dysbiosis has a negative impact on cognition [55]. Gut microbiota interventions improve cognitive performance, in terms of visuospatial memory, verbal learning and memory, and aspects of attentional vigilance [56].

**Figure 4:**
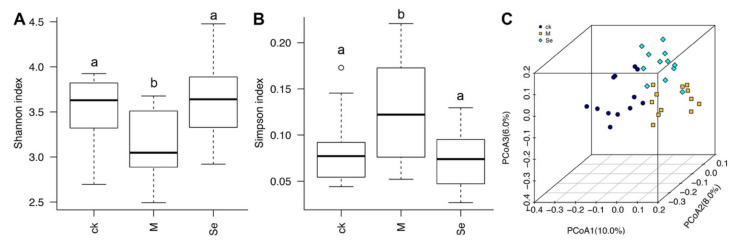
Effects of SeMet on the (**A**,**B**) α-diversity and (**C**) β-diversity of gut microbiota in mice. The same letter within each column indicates no significant difference (*p* > 0.05). CK is short for the control group, M is short for the model group, and Se is short for the SeMet-treated group.

D-Galactose decreased the α-diversity of gut microbiota, and SeMet reversed D-galactose-induced reduction of α-diversity (Figure 4A,B). β-Diversity analysis revealed that dots presented each group was isolated from each other, suggesting the structures of gut microbiota in the three groups were different (Figure 4C). A former study indicated that there were correlations between microbiota diversity and enhanced cognitive flexibility and executive function [56]. Our result implies that SeMet might improve cognitive impairment by modulating the diversity of gut microbiota.

**Figure 5:**
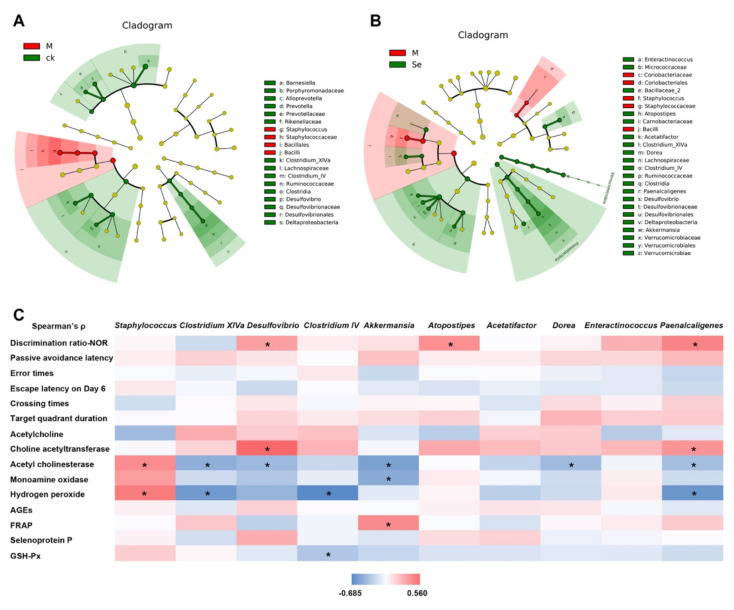
Effects of SeMet on the taxonomic structure of gut microbiota. (**A**) Statistically differential taxa between the control (CK) group and model (M) group. (**B**) Statistically differential taxa between the model (M) group and SeMet group (Se). (**C**) Correlation between certain taxa and behavioral or physiological parameter. * indicates significant difference (*p* < 0.05). NOR is short for novel object recognition. AGEs is short for advanced glycation end products. FRAP is short for ferric ion reducing antioxidant power. GSH-Px is short for glutathione peroxidase.

Taxonomic structure analysis revealed that at the genus level, *Staphylococcus* was enriched while *Clostridium IV*, *Clostridium XIVa*, and *Desulfovibrio* were inhibited in the model group (Figure 5A). SeMet successfully reversed D-galactose-induced changes of these genus (Figure 5B). Compared with the model group, the relative abundances of *Akkermansia*, *Dorea*, *Acetatifactor*, *Atopostipes*, *Enteractinococcus*, and *Paenalcaligenes* were also increased in the SeMet group. Correlations between certain taxa and behavioral or physiological parameters were observed (Figure 5C).

*Staphylococcus* belongs to the *Bacilli* class, *Staphylococcaceae* family. An elevated relative abundance of *Staphylococcus* was found in the feces of patients with Alzheimer′s disease compared with that in healthy volunteers [57]. Some strains of *Staphylococcus* secrete toxins and injure the intestinal barrier function. Impaired intestinal barrier function allows excessive entrance of harmful bacterial metabolites [58]. These metabolites disrupt the blood-brain barrier, pass through it, and induce inflammation and oxidative stress in the central nervous system, thereby causing cognitive impairment. *Clostridium IV* and *Clostridium XIVa*, both of which belong to the *Clostridia* class, are known as short-chain fatty acids (SCFAs) producers. SCFAs enhance the intestinal barrier function. Based on our results, the relative abundance of *Staphylococcus* was positively correlated to the hippocampal H_2_O_2_ level, while the relative abundances of *Clostridium IV* and *Clostridium XIVa* were negatively correlated to the hippocampal H_2_O_2_ level (Figure 5C). It hinted that SeMet might protect the hippocampus from oxidative stress-induced damage by regulating the three genera and improving the intestinal barrier.

*Desulfovibrio*, belongs to the *Deltaproteobacteria* class, *Desulfovibrionaceae* family. Xu reported that D-galactose caused a decrease of *Desulfovibrio* in mice and the cognition of mice was improved together with the increase of *Desulfovibrio* [59]. Sodium oligoarginine (GV-971), a new AD drug approved by China FDA which targeted the gut microbiota to improve cognitive function of mild to moderate AD patients, showed the enrichment of *Desulfovibrionaceae* [60]. The relative abundance of *Desulfovibrio* was positively associated with the discrimination ratio and hippocampal ChAT activity, while negatively associated with the AchE activity in this study (Figure 5C). It was reported that *Desulfovibrio* produced hydrogen sulfide. Hydrogen sulfide acts as a novel neuro-modulator and neuroprotective agent, which effectively improves cognition impairment in several animal models, such as Alzheimer’s model mice and rats and diabetic rats [61,62]. It was possible that SeMet enriched *Desulfovibrio*, increased the hydrogen sulfide level, and regulated the neurotransmitter system to ameliorate the cognitive impairment.

*Akkermansia* belongs to the *Verrucomicrobiae* class, *Verrucomicrobiaceae* family. It is a probiotic. Recent studies revealed that it benefited cognition. It promoted the reduction of Aβ 40–42 levels in the cerebral cortex and relieved impairment of spatial learning and memory in APP/PS1 Alzheimer’s disease model mice [63]. It reversed the high-fat, high-cholesterol-diet-induced cognitive dysfunction and restored brain metabolism in rats [64]. Previous studies showed dietary Se increased the relative abundance of *Akkermansia* [19,65]. Similar phenomenon was observed in this study (Figure 5B). Correlation analysis indicated that the relative abundance of *Akkermansia* was negatively associated with the AchE and MAO activity, while positively associated with FRAP (Figure 5C), suggesting *Akkermansia* might play a role in improving cognitive performance by regulating the neurotransmitter system and increasing the antioxidant activity in hippocampus.

**Figure 6:**
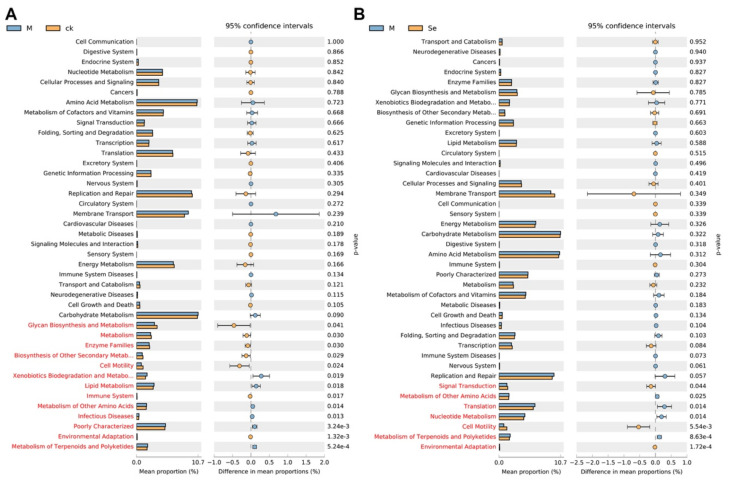
Effects of SeMet on the predicted functions of gut microbiota. (**A**) Differential predicted functions between the control (CK) group and the model (M) group. (**B**) Differential predicted functions between the M group and the SeMet (Se) group. Statistically differential predicted functions are marked in red.

Functional prediction based on the phylo-genetic investigation of gut microbiota was conducted to investigate the differential functions of gut microbiota between groups (Figure 6A,B). The results suggested that the relative abundance of genes associated with the metabolism of other amino acids, and metabolism of terpenoids and polyketides were upregulated in the model group and the relative abundance of genes associated with cell motility and environmental adaptation were downregulated in the model group. SeMet effectively reversed the alterations. Further experiments (e.g., untargeted analysis of the intestinal content) are needed to testify whether these changes did occur in the gut. A previous study suggested that fecal metabolites which were associated with neurosubstances, vitamins, amino acids, fatty acids, incretions, carbohydrate metabolism, nucleic acid metabolism, lipid metabolism, and bile acid metabolism were altered in normal mice supplemented with SeMet [66]. Notably, the levels of neurosubstances (e.g., serotonin and melatonin), which were known to be involved in the gut-brain axis, were reduced in Se-deficient mice compared with that in SeMet-supplemented mice. However, it still remains unclear whether SeMet causes similar changes in D-galactose-treated mice.

## 4. Conclusions

SeMet effectively ameliorated D-galactose-induced cognitive impairment, including recognition memory, fear-motivated memory, and spatial memory. The performance of mice in the SeMet group in the novel object recognition test, passive avoidance task, and Morris water maze test was much better than that of mice in the model group. Attenuating neurotransmitter dysregulation, decreasing oxidative stress, and alleviating dysbiosis may be involved in the strategies of SeMet. D-Galactose-induced decrease of Ach in the hippocampus was reversed in the SeMet group, via elevating the ChAT activity and inhibiting the AchE activity. D-Galactose-induced accumulation of oxidative stress-related products in the hippocampus, including H_2_O_2_ and AGEs, was decreased in the SeMet group, while the hippocampal antioxidant activity and SelP level were increased. D-Galactose-induced dysbiosis was attenuated in the SeMet group by elevating the α-diversity and modulating the taxonomic structure. Our findings suggest that SeMet has the potential to be used to attenuate age-related cognitive impairment and probably can be applied in the prevention or treatment of neurodegenerative diseases.

## Data Availability

Not applicable.
